# Foreword to volume 3, issue 6

**DOI:** 10.1002/mgg3.185

**Published:** 2015-11-11

**Authors:** P. Suzanne Hart, Maximilian Muenke

## Abstract

As 2015 draws to a close so too do the many celebrations of the 150th anniversary of Mendel's presentation of his work entitled “Experiments in Plant Hybridization” to the Natural History Society of Brno.

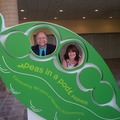

As 2015 draws to a close so too do the many celebrations of the 150th anniversary of Mendel's presentation of his work entitled “Experiments in Plant Hybridization” to the Natural History Society of Brno. His handwritten manuscript is shown in Figure 1. His findings were published in 1866 in the Society's journal (Fig. 2). *MGGM* marked the beginning of the year with an invited commentary by Drs. John Opitz and Diana Bianchi reflecting on Mendel and his accomplishments (Opitz and Bianchi 2015). Additional reflections were published this year in other journals (e.g., Birchler 2015; Chadov et al. 2015; Matalová and Matalová 2015; Singh 2015). Various celebrations were held around the world, including one at the Old Brno Abbey where Mendel carried out his famous experiments. The *MGGM* editor, Dr. Max Muenke, hosted two parties to mark the occasion. The first was held in Maryland and featured several types of pea soup. A presentation on Mendel's life and work was presented by one of the Clinical Genetics residents, Dr. Mauricio De Castro. Dr. De Castro's Invited Commentary, which includes information on Mendel's scientific work outside of genetics, will be published in the January 2016 issue of *MGGM*. Max Muenke also hosted a party in Freiberg, Germany where the guest of honor was Mendel's great‐great‐grandnephew, Father Clemens Richter. This issue of the journal contains an Invited Commentary by Father Clemens reflecting on the life of his famous uncle. The 2015 American Society of Human Genetics annual meeting, held in Baltimore MD in October, had an exhibit dedicated to the 150th anniversary. The exhibit from the Mendel Museum of Mararyk University, Brno featured informative, hands‐on exhibits. Figure 3 shows us enjoying the festivities at the meeting. As Mendel said “My scientific studies have afforded me great gratification; and I am convinced that it will not be long before the whole world acknowledges the results of my work.” At *MGGM*, we are happy to help acknowledge Mendel's legacy and contributions to science.

**Figure 1:**
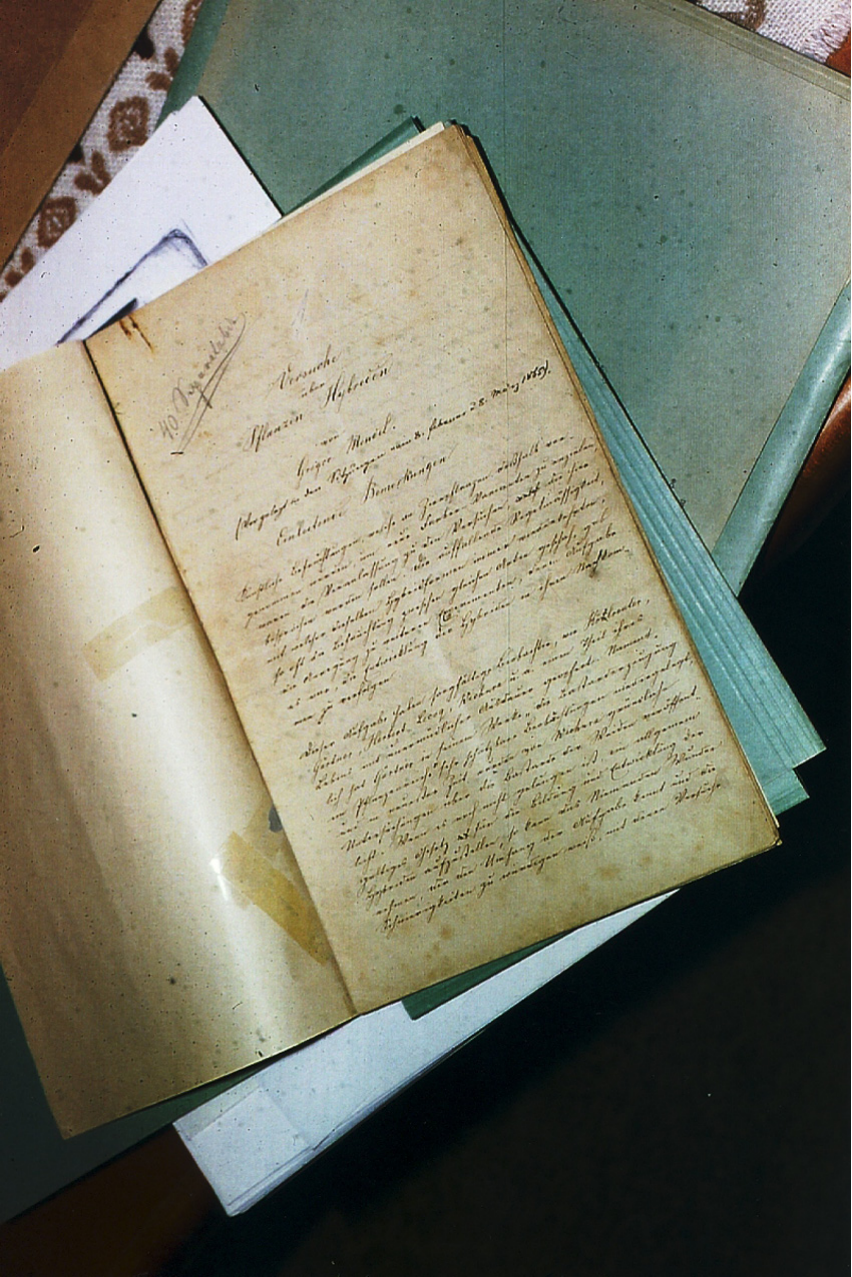
Mendel's handwritten manuscript. Photo courtesy of Father Clemens and Roger Stevenson.

**Figure 2:**
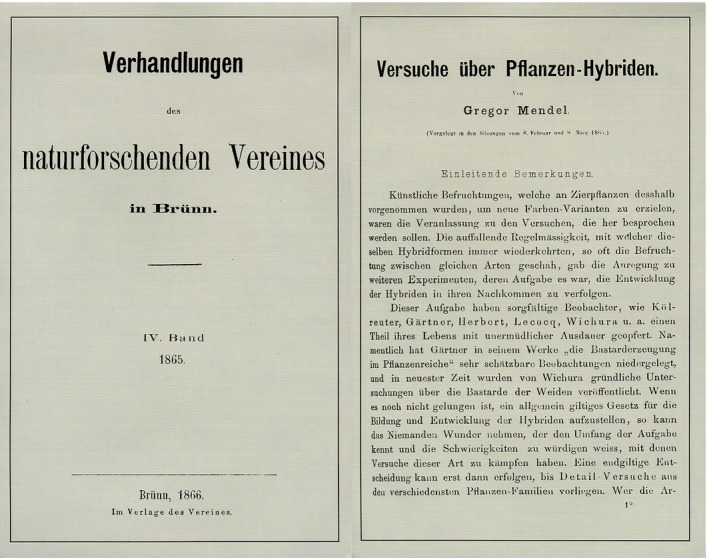
Mendel's original publication.

**Figure 3:**
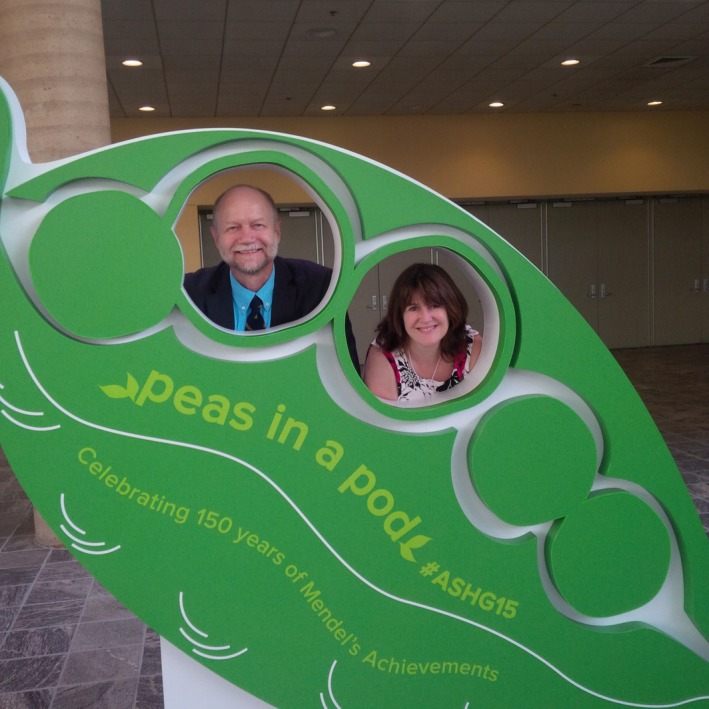
Max Muenke and Suzanne Hart enjoying the 150 years of Mendel exhibit at the 2015 Annual Meeting of the American Society of Human Genetics.

## References

[mgg3185-bib-0001] Birchler, J. A. 2015 Mendel, mechanism, models, marketing and more. Cell 163:9–11. 26406361 10.1016/j.cell.2015.09.008

[mgg3185-bib-0002] Chadov, B. F. , N. B. Fedorova , and E. V. Chadova . 2015 Conditional mutations in *Drosophilia melanogaster*: on the occasion of the 150th anniversary of G. Mendel's report in Brünn. Mutat. Res. Rev. Mutat. Res. 765:40–55. 26281767 10.1016/j.mrrev.2015.06.001

[mgg3185-bib-0003] Matalová, A. , and E. Matalová . 2015 Plant genetics: Czech centre marks Mendel anniversary. Nature 518:303. 25693553 10.1038/518303e

[mgg3185-bib-0004] Mendel, J. G. 1866 Versuche über Pflanzen‐hybriden Verhandlungen des naturforschenden Vereines in Brünn, Bd. IV für das Jahr, 1865 Abhandlungen: 3–47.

[mgg3185-bib-0005] Opitz, J. M. , and D. W. Bianchi . 2015 MENDEL: Morphologist and Mathematician Founder of Genetics – To Begin a Celebration of the 2015 Sesquicentennial of Mendel's Presentation in 1865 of his *Versuche über Pflanzenhybriden* . Mol. Genet. Genomic Med. 3:1–7. doi:10.1002/mgg3.127. 25629074 10.1002/mgg3.127 PMC4299709

[mgg3185-bib-0006] Singh, R. S. 2015 Limits of imagination: the 150th anniversary of Mendel's laws and why Mendel failed to see the importance of his discovery for Darwin's theory of evolution. Genome 15:1–7. 10.1139/gen-2015-0107 26372894

